# Rejuvenated endothelial progenitor cells through overexpression of cellular prion protein effectively salvaged the critical limb ischemia in rats with preexisting chronic kidney disease

**DOI:** 10.1186/s13287-022-03119-0

**Published:** 2022-09-02

**Authors:** Jui-Po Yeh, Pei‐Hsun Sung, John Y. Chiang, Chi-Ruei Huang, Yi-Ling Chen, Jui-Pin Lai, Jiunn-Jye Sheu

**Affiliations:** 1 Department of Plastic and Reconstructive Surgery, Kaohsiung Chang Gung Memorial Hospital, Chang Gung University College of Medicine, 123, Dapi Road, Niaosung Dist., Kaohsiung City, 833253 Taiwan; 2 Division of Cardiology, Department of Internal Medicine, Kaohsiung Chang Gung Memorial Hospital, Chang Gung University College of Medicine, Kaohsiung, 833253 Taiwan; 3 Center for Shockwave Medicine and Tissue Engineering, Kaohsiung Chang Gung Memorial Hospital, Kaohsiung, 833253 Taiwan; 4 Institute for Translational Research in Biomedicine, Kaohsiung Chang Gung Memorial Hospital, Kaohsiung, 833253 Taiwan; 5 Department of Computer Science and Engineering, National Sun Yat-Sen University, Kaohsiung, 804201 Taiwan; 6 Department of Healthcare Administration and Medical Informatics, Kaohsiung Medical University, Kaohsiung, 807378 Taiwan; 7 Division of Thoracic and Cardiovascular Surgery, Department of Surgery, Kaohsiung Chang Gung Memorial Hospital, Chang Gung University College of Medicine, 123, Dapi Road, Niaosung Dist., Kaohsiung, 83301 Taiwan

**Keywords:** Endothelial progenitor cells, Chronic kidney disease, Critical limb ischemia, Rejuvenation

## Abstract

**Background:**

This study tested the hypothesis that overexpression of cellular prion protein in endothelial progenitor cells (PrPc^OE^-EPCs), defined as “rejuvenated EPCs,” was superior to EPCs for salvaging the critical limb ischemia (CLI) induced after 28-day chronic kidney disease (CKD) induction in rat.

**Methods and Results:**

Cell viability and flow cytometric analyses of early/late apoptosis/total-intracellular ROS/cell cycle (sub-G1, G2/M phase) were significantly higher in EPCs + H_2_O_2_ than in EPCs that were significantly reversed in PrPc^OE^-EPCs + H_2_O_2_ (all *p* < 0.001). The protein expressions of inflammation (IL-1ß/IL-6/MMP-9/p-NF-κB) were significantly increased in EPC + TNF-α than in EPCs that were significantly reversed in PrPc^OE^-EPCs + TNF-α (all *p* < 0.001). Adult-male SD rats (*n* = 8/each group) were categorized into group 1 (sham-operated control), group 2 (CKD + CLI), group 3 [CKD + CLI + EPCs by intravenous (0.6 × 10^5^)/intra-muscular (0.6 × 10^5^) injections at 3 h after CLI induction], group 4 (CKD + CLI + PrPc^OE^-EPCs/dose-administration as group 3) and group 5 (CKD + CLI + si*Prnp*-EPCs/dose-administration as group 3). By day 14 after CLI induction, the ratio of ischemia to normal blood flow (INBF) in CLI area was highest in group 1/lowest in group 2/significantly higher in group 4 than in groups 3/5 and significantly higher in group 3 than in group 5 (all *p* < 0.0001). Histopathology demonstrated that the angiogenesis (number of small vessels/CD31 + cells) exhibited a similar trend, whereas the fibrosis/kidney injury score exhibited an opposite pattern of INBF among the groups (all *p* < 0.0001). The protein expressions of angiogenesis (SDF-1α/VEGF/CXCR4)/cell-stress signaling (p-PI3K/p-Akt/p-m-TOR) were significantly and progressively increased from groups 1–4 that were reversed in group 5 (all *p* < 0.0001). The protein expressions of fibrotic (p-Smad3/TGF-ß)/oxidative-stress (NOX-1/NOX-2/oxidized-protein)/apoptotic (mitochondrial-Bax/cleaved caspase3/cleaved PARP)/mitochondrial-damaged (cytosolic-cytochrome-C) biomarkers displayed an opposite pattern of INBF among the groups (all *p* < 0.0001).

**Conclusion:**

PrPc^OE^-EPCs were superior to EPCs only therapy for salvaging the CLI.

**Supplementary Information:**

The online version contains supplementary material available at 10.1186/s13287-022-03119-0.

## Introduction

Atherosclerotic peripheral arterial occlusive disease (PAOD), one of the major manifestations of systemic atherosclerosis [1], has been identified to affect 12% of the adult population and up to 20% of the elderly [2]. Patients with PAOD may frequently develop critical limb ischemia (CLI) at the late stage of the disease [2, 3]. Undoubtedly, the CLI commonly occurs when arterial blood flow is restricted so severely that perfusion of capillary beds is inadequate to sustain tissue viability [4, 5]. Importantly, researches have established that numerous PAOD patients are asymptomatic prior to the development of CLI [6, 7] which poses as an obstacle to early diagnosis and early treatment for the purposes of slowing or abolishing disease progression and development of complications.

Previous studies in epidemiology have clearly identified that chronic kidney disease (CKD) patients are at a higher risk of developing PAOD and its adverse clinical outcomes are much higher than individuals in the general population who have normal renal function [8–12]. Of importance is that while CKD and PAOD share common predisposing factors, emerging studies indicate that their coexistence is not merely an association; instead, CKD represents a strong, independent risk factor for the propagation and poorer prognostic outcome of PAOD patients [13, 14]. Furthermore, a clinical study with a large sample size has established that the incidence of CKD in PAOD patients has been reported to 26% in stage 2, 47% in stage 3, 11% in stage 4 and 17% in stage 5 and those with CKD had notably higher incidence of coronary artery disease (i.e., 1.8-fold higher) [15]. The clinical observational studies have further identified that in-hospital and long-term mortality, morbidity, amputation rates, duration and costs of hospitalization and in-hospital treatment, and complications in patients with combined CKD and PAOD are extremely high [15, 16], suggesting that despite the advent of screening [15, 16] and interventional procedures (i.e., bypass surgery or endovascular treatment) [8, 16–18], the long-term clinical outcomes remain suboptimal, especially in patients with CLI [16–18]. These issues [8, 11–18] raise the need of considering a safe and effective alternative treatment for PAOD patients with coexistence of CKD setting.

The therapeutic effect of endothelial progenitor cells (EPCs) on rescuing the PAOD/CLI is currently contradictory because the EPCs function is always impaired in CKD and PAOD settings [19–21]. Accordingly, prior to treatment of PAOD/CLI, the improvement of EPCs function for angiogenesis/neovascularization, resulting in restoring the blood flow in the ischemic region, is extremely important. Initially, cellular prion protein (PrPc) was identified as a glycosylphosphatidylinositol-anchored glycoprotein that is mainly expressed in the central nervous system and nerve cells and exists in other tissues [22, 23]. Later, the PrPc has been identified to play a crucial protein on neuroprotection and serve as an essential survival role due to the retardation of Bcl-2-associated protein X (Bax)-mediated cell death [24]. Besides, some basic researches have displayed that PrPc-null cells are more susceptible to serum deprivation and oxidative stress than PrPc-expressing cells [25, 26]. Our recent study has further demonstrated that upregulation of PrPc in adipose-derived mesenchymal stem cells (ADMSCs) effectively preserved the residual renal function in CKD rats [27]. The aforementioned issues [19–27] raise our hypothesis that enhancing the expression of PrPc in EPCs might play an essential role on the “Rejuvenation of EPCs” for improving the ability of EPC proliferation and angiogenesis as well as restoring blood flow in the ischemic area, resulting in the salvage of the CLI.

## Materials and methods

### Ethics

All animal procedures were approved by the Institute of Animal Care and Use Committee at Kaohsiung Chang Gung Memorial Hospital (Affidavit of Approval of Animal Use Protocol No. 2019102404) and performed in accordance with the Guide for the Care and Use of Laboratory Animals. Animals were housed in an Association for Assessment and Accreditation of Laboratory Animal Care International (AAALAC; Frederick, MD, USA)-approved animal facility in our hospital with controlled temperature and light cycles (24 °C and 12/12 light cycle).

### Transfection of EPCs with plasmids for PrPc expression

The procedure and protocol have been reported by our recent studies [28, 29]. The pCS6-PRNP plasmid was purchased from Transomic Technologies (Huntsville, Alabama, USA). Briefly, the plasmid transfection process was carried out with Lipofectamine 3000 (Invitrogen, Life technologies, Carlsbad, CA, USA) according to the manufacturer’s instructions with slight modifications. Cells were replated 24 h before transfection at a density of 5 × 10^5^ cells in 4 ml of fresh culture medium in a 6-cm plastic dish. The steps were briefly described as follows: 10 μg PRNP expression vector and 20 μl Lipofectamine 3000 were first incubated at room temperature for 15 min, followed by overnight incubation of cells at 37 °C in a humidified atmosphere of 5% CO_2_ and Lipofectamine (i.e., mixed them together), and then, relevant experiments were carried out.

### Transfection of cells with siRNA

Transient transfection of cells with siRNA was performed with Lipofectamine RNAiMAX (Invitrogen, Life technologies, Carlsbad, CA, USA) according to the manufacturer’s instructions with slight modifications. Briefly, 1 × 10^6^ cells were seeded to 10-cm plastic dish overnight. For use in transfection, Lipofectamine RNAiMAX was incubated with 100 pmol of si*Prnp* at room temperature for 15 min. The sequence of si*Prnp* is 5’-GCCCUCUUUGUGACUACAUTT-3’. Cells were incubated with siRNA complex at 37 °C in a humidified atmosphere of 5% CO_2_ before being harvested.

### MTT cell viability assay

The growth of circulatory EPCs derived from rats was determined by the MTT assay. About 2 × 10^3^ cells in 100 μL of medium (stock in 100% EtOH at 100 mM concentration, working in 1 mM) were seeded into wells of a 96-well plate and incubated for a duration of 6 h. Then, the medium was changed, followed by incubation for an additional 24 h to 72 h. For MTT assay, 2000 cells per well were seeded in 96 wells in 100 μL of medium with or without 50 µM H_2_O_2_ for 24 h (i.e., oxidative stress tests). At the time point for detection, the medium was removed, and 200 μl MTT reagent was added to the cells for 30 min. After incubation, the purple crystal sediment was dissolved in DMSO and read at 540 nm in an ELISA reader. The absorbance value was used to represent the cell number.

### Flow cytometric analysis for determining the cellular apoptosis, fluorescent intensity of reactive oxygen species (ROS) and cell cycle

The procedure and protocol for determining the circulating level of mononuclear cells have been reported in detail in our previous study [30]. Briefly, Annexin V kit (Pharmingen, Becton Dickinson, San José, CA, USA) was used for apoptosis analysis according to the manufacturer’s instructions. The supernatant was decanted and the pellet was resuspended in 200 μL of 1 × annexin V-binding buffer. Cells were incubated with 2 μL of annexin V-FITC and 5 μL of propidium iodide (PI) for 15 min at room temperature in the dark. Finally, 500 μL of 1 × annexin V-binding buffer was added, followed by immediate flow cytometric analysis [i.e., the percentages of viable and apoptotic cells were determined by flow cytometry using double staining of annexin V and propidium iodide (PI)]. This is a simple and popular method for the identification of apoptotic cells (i.e., early [annexin V +/PI −] and late [annexin V + /PI +] phases of apoptosis).

For assessment of total intracellular ROS, the cells were incubated with a serum-free medium containing 10 μM H_2_DCFDA in a 37 °C incubator for 20 min just after the cells were rinsed twice with PBS. After rinsing twice with PBS to remove residual H_2_DCFDA, the cells were incubated with a serum-containing culture medium for an additional 30 min. Following trypsinization, the cells were suspended in PBS and analyzed by flow cytometry in the FL1 channel.

For cell cycle staining, the cells were harvested by trypsinization and neutralized with a serum-containing medium after well preparation. In detail, after rinsing the cells twice with 4 ml of PBS, the PBS was removed after centrifugation at 1000 g for 5 min and 1.0 ml of the PBS was retained. To avoid cell agglutination, the cell suspension was continuously shaken on a vortex, and 3 ml of pure ethanol was slowly dropped into the cell suspension to reach the final concentration 75%. Finally, the cell suspension was placed at − 20 °C for at least 24 h.

### Collection of the peripheral blood from animals for culturing mononuclear cell-derived EPCs for further individual study

The procedure and protocol have been thoroughly described in our previous studies [31, 32]. Briefly, healthy rats (i.e., for in vitro studies) and CKD rats (i.e., autologous EPCs treatment) were anesthetized with inhalational 2.0% isoflurane (i.e., CKD rats at day 7 after CKD induction for CLI treatment) for the collection of 3 mL of peripheral blood from tail vein. The isolated mononuclear cells from peripheral blood were cultured in a 100 mm diameter dish with 10 mL DMEM culture medium containing 10% FBS. By 21-day culturing, abundant EPCs were obtained from culturing. Flow cytometric analysis was performed for identification of cellular characteristics (i.e., EPC surface markers) after cell labeling with appropriate antibodies on day 21 of cell cultivation prior to autologous EPC treatment for CLI rats.

### Flow cytometric analysis for assessment of culturing EPCs based on surface markers

For characterizing the EPCs surface markers after collecting from the cell culture, the EPCs were immunostained for 30 min on ice with the following antibodies: PE-conjugated antibodies against CD133 (BD Pharmingen), Sca-1 (BD Pharmingen), and CD34 (BD Pharmingen); Fluorescein isothiocyanate (FITC)-conjugated antibodies against c-kit (BD Pharmingen); Monoclonal antibodies against CD31 (Abcam), VEGF (Abcam), KDR (Thermo); and vascular endothelial cadherin (Ve-Cad) (Abcam). Cells labeled with non-fluorescence-conjugated antibodies were further incubated with Alexa Fluor 488-conjugated antibodies specifically against mouse or rabbit IgG (Invitrogen). Isotype-identical antibodies (IgG) served as controls. Flow cytometric analyses were performed by utilizing a fluorescence-activated cell sorter (Beckman Coulter FC500 flow cytometer). The results showed that c-Kit +/CD31 + , Sca-1 +/CD31 + , vascular endothelial cadherin (Ve-Cad) +/CD34 + and KDR +/CD34 + cells were the four dominant EPCs to be utilized together in the current study.

### Evaluating angiogenesis using Matrigel assay

The overexpression of PrP^C^ in EPCs, i.e., called PrPc^OE^-EPCs, was defined as “rejuvenation of EPCs.” EPCs and PrPc^OE^-EPCs were plated in 96-well plates at 1.0 × 10^4^ cells/well in 150 µL serum-free M199 culture medium mixed with 50 µL cold Matrigel (Chemicon International, Inc. Temecula, CA, USA) for 24 h using passage 3–4 EPCs incubated at 37 °C in 5% CO_2_. Three random microscopic images (200 ×) were taken from each well to count cluster, tube and network formations, and the mean values were obtained. Additionally, both cumulative and mean tube lengths will be calculated by Image-Pro Plus software (Media Cybernetics, Bethesda, MD, USA).

### Animal models of CKD and CLI and animal grouping

The protocol and procedure of CKD induction have been reported in our previous studies in detail [27, 32]. Briefly, pathogen-free, adult-male Sprague Dawley rats (Charles River Technology, BioLASCO) were anesthetized with 2.0% inhalational isoflurane for midline laparotomies. The sham-operated control (SC) group received laparotomy only, while CKD induction was performed in the other groups. CKD was induced by right nephrectomy plus arterial ligation of upper and middle thirds of blood supply to the left kidney, thereby creating a 5/6 nephrectomy model with a limited kidney function.

The procedure and protocol of CLI were based on our previous report [33]. Briefly, male SD rats in CLI groups were anesthetized by inhalation of 2.0% isoflurane. The rats were placed in a supine position on a warming pad at 37 °C with the left hind limb shaved. Under sterile conditions, the left femoral artery, small arterioles, circumferential femoral artery and veins were exposed and ligated over their proximal and distal portions before removal. To avoid the presence of collateral circulation, the branches were removed altogether. For animals serving as controls, the arteries were only isolated without ligation.

Adult-male rats (*n* = 8 for each group) were categorized into group 1 (sham-operated control), group 2 (CKD + CLI), group 3 [CKD + CLI + EPCs by intravenous (0.6 × 10^5^ cells) and intra-muscular (0.6 × 10^5^ cells) injections at 3 h after CLI induction], group 4 (CKD + CLI + PrPc^OE^-EPCs with dose and administration route as group 3) and group 5 (CKD + CLI + si*Prnp*-EPCs with dose and administration route as group 3).

### Examination of time courses of plasma level of creatinine, blood urine nitrogen (BUN) and collection of 24 h urine for the ratio of urine protein to urine creatinine at baseline and days 14 and 28 after CKD induction.

Blood samples were collected from all animals in each group to measure changes in plasma creatinine level prior to, and at days 14 and 28 after CKD induction. The procedure and protocol for determining the ratio of urine protein to urine creatinine were based on our previous reports [27, 32]. To collect 24 h urine for individual study, each animal was placed into a metabolic cage [DXL-D, space: 19 × 29 × 55 cm, Suzhou Fengshi Laboratory Animal Equipment, China] for 24 h with free access to food and water. Urine at 24 h was collected in all animals prior to and by days 14, 28 and 42 (i.e., a 24 h-interval for every urine collection) after the CKD procedure to determine the ratio of urine protein to urine creatinine.

### Measurement of blood flow with laser Doppler

The procedure and protocol were based on our previous report [33]. Briefly, animals were anesthetized by inhalation of isoflurane (2.0%) prior to CKD induction and at days 1, 7 and 14 after CLI induction prior to being euthanized. The rats were placed supine on a warming pad (37 °C), and blood flow was detected in both inguinal areas by a laser Doppler scanner (moorLDLS, Moor Instruments, UK). The ratio of flow in the left (ischemic) and right (normal) legs was computed. On day 14 after CLI induction (at the end of study period, i.e., by day 42 after CKD induction), the animals were euthanized and the quadriceps muscles were collected for individual study.

### Qualitative analysis of kidney injury score at day 42 after CKD induction

In detail, the histopathologic scoring of kidney injury was assessed in a blinded fashion as we previously reported [27, 32]. Briefly, kidney specimens from all animals were fixed in 10% buffered formalin, embedded in paraffin, sectioned at 5 μm and stained with hematoxylin and eosin (H&E) for light microscopy. The scoring system reflected the grading of tubular necrosis, loss of brush border, cast formation, Bowman's capsule and tubular dilatation in 10 randomly chosen, non-overlapping fields (200x) as follows: 0 (none), 1 (≤ 10%), 2 (11–25%), 3 (26–45%), 4 (46–75%) and 5 (≥ 76%).

### Immunohistochemical (IHC) and immunofluorescent (IF) staining by day 42 after CKD induction

The procedure and protocol for IHC and IF staining have been described in our previous reports [27–32]. For IHC and IF staining, rehydrated paraffin sections were first treated with 3% H_2_O_2_ and incubated with Immuno-Block reagent (BioSB, Santa Barbara, CA, USA) for 30 min at room temperature. Sections were then incubated with primary antibodies specifically against zonula occludens-1 (ZO-1) (1: 200, Abcam), kidney injury molecule (KIM)-1 (1: 400, Novus), synaptopodin (1:500, Santa Cruz) and alpha-smooth muscle actin (α-SMA) (A2547, 1: 500, Sigma-Aldrich), while sections incubated with the use of irrelevant antibodies served as controls. Three sections of kidney specimen and quadriceps muscle from each rat were analyzed. For quantification, three random chosen HPFs (200 × or 400 × for IHC and IF studies) were analyzed in each section. The mean number of positively stained cells per HPF for each animal was then determined by summation of all numbers divided by 9.

An IF-based scoring system was adopted for semi-quantitative analysis of KIM-1 in the kidney as a percentage of positive cells in a blinded fashion (score of positively stained cell for these biomarkers as: 0 = negative staining; 1 = < 15%; 2 = 16–25%; 3 = 26–50%; 4 = 51–75%; 5 = 76–100% per HPF).

### Western blot analysis by day 42 after CKD induction

The procedure and protocol for Western blot analysis have been described in our previous reports [27–32]. Briefly, equal amounts (50 μg) of protein extracts were loaded and separated by SDS-PAGE using acrylamide gradients. After electrophoresis, the separated proteins were transferred electrophoretically to a polyvinylidene difluoride (PVDF) membrane (GE, UK). Nonspecific sites were blocked by incubation of the membrane in blocking buffer [5% nonfat dry milk in T-TBS (TBS containing 0.05% Tween 20)] overnight. The membranes were incubated with the indicated primary antibodies [PrPc (1: 1000, Abcam), phosphorylated (p)-PI3K (1: 1000, Cell Signaling), PI3K (1: 1000, Cell Signaling), p-Akt (1: 1000, Cell Signaling), Akt (1: 1000, Cell Signaling), p-m-TOR (1: 1000, Cell Signaling), m-TOR (1: 1000, Cell Signaling), NOX-1 (1: 1000, Sigma), NOX-2 (1: 1000, Sigma), cytosolic (1: 2000, BD) and mitochondrial (1: 2000, BD) cytochrome C, mitochondrial Bax (1: 1000, Abcam), cleaved caspase 3 (1: 1000, Cell Signaling), cleaved PARP (1: 1000, Cell Signaling), transforming growth factor beta (TGF-ß) (1: 1000, Abcam), *p*-endothelial nitric oxide synthase (eNOS) (1: 1000, Abcam), CD31 (1: 1000, Abcam), vascular endothelial growth factor (VEGF) (1: 1000, Abcam), stromal cell-derived growth factor (SDF)-1α (1: 1000, Cell Signaling), CXCR4 (1: 1000, Abcam), p-Smad3(Ser423/425) (1: 1000, Cell Signaling) and Actin (1: 10,000, Chemicon)] for 1 h at room temperature. Horseradish peroxidase-conjugated anti-rabbit immunoglobulin IgG (1:2000, Cell Signaling, Danvers, MA, USA) was used as a secondary antibody for one-hour incubation at room temperature. The washing procedure was repeated eight times within one hour. Immunoreactive bands were visualized by enhanced chemiluminescence (ECL; Amersham Biosciences, Amersham, UK) and exposed to Biomax L film (Kodak, Rochester, NY, USA). For quantification, ECL signals were digitized using Labwork software (UVP, Waltham, MA, USA).

### Histological quantification of quadriceps muscle and kidney fibrosis

The procedure and protocol have been described in detail in our previous reports [27, 32, 33]. Briefly, hematoxylin and eosin (H&E) and Masson's trichrome staining were used to identify fibrosis in kidney and quadriceps muscle, respectively. Three serial sections of these specimens in each animal were prepared at 4 µm thickness by Cryostat (Leica CM3050S). The integrated area (µm^2^) of fibrosis on each section was calculated using the Image Tool 3 (IT3) image analysis software (University of Texas, Health Science Center, San Antonio, UTHSCSA; Image Tool for Windows, Version 3.0, USA). Three randomly selected high-power fields (HPFs) (100×) were analyzed in each section. After determining the number of pixels in each infarct and fibrotic area per HPF, the numbers of pixels obtained from three HPFs were calculated. The procedure was repeated in two other sections for each animal. The mean pixel number per HPF for each animal was then determined by calculating all pixel numbers and divided by 9. The mean integrated area (µm^2^) of fibrosis in quadriceps muscle and kidney per HPF was obtained using a conversion factor of 19.24 (since 1 µm^2^ corresponds to 19.24 pixels).

### Statistical analysis

Quantitative data were expressed as mean ± standard deviation. Statistical analysis was adequately performed by ANOVA followed by Bonferroni multiple comparison post hoc test. Statistical analysis was performed using SPSS statistical software for Windows version 22 (SPSS for Windows, version 22; SPSS, IL, USA). A value of *p* < 0.05 was considered as statistically significant.

## Results

### Impact of PrPc^OE^ on cell viability and cell cycle (Figure 1)

First, to elucidate the cell viability, the circulatory EPCs derived from healthy rat were categorized into E1 (EPCs only), E2 (EPCs + H_2_O_2_) and E3 (PrPc^OE^-EPCs + H_2_O_2_). The result of MTT assay demonstrated that the cell viability (i.e., proliferation rate) was significantly lower in E2 than in E1 and E3 and significantly higher in E1 than in E3 at time points of 24, 48 and 72 h, respectively.

Second, we assessed the impact of PrPc overexpression in EPCs (i.e., PrPc^OE^-EPCs) on cell cycle activity. The cells were categorized as the aforementioned E1–E3. The flow cytometric analysis showed that the Sub-G1 phase of cell cycle, an indicator of apoptosis, was significantly higher in E2 than E1 and E3, and significantly higher in E3 than in E1. On the other hand, the G1 phase of cell cycle, an indicator of the cell synthesis of mRNA and proteins in preparation for subsequent steps for cell division, exhibited an opposite pattern of Sub-G1 phase among the three groups. The *S* phase of cell cycle, an indicator of cell synthesis responsible for the synthesis or replication of DNA, was significantly higher in E3 than in E1 and E2, and significantly higher in E1 than E2, suggesting that PrPc^OE^ augmented the EPCs division in cell mitosis phase. The G2/M phase, an indicator of mitosis, was significantly higher in E2 than in E1 and E3, significantly higher in E3 than in E1, suggesting that H_2_O_2_-induced G2/M phase at an arrest stage was reversed by PrPc^OE^.

### Impact of PrPc^OE^ on inhibiting the intracellular reactive oxygen species (ROS) and cell apoptosis (Figure 2)

**Fig. 1:**
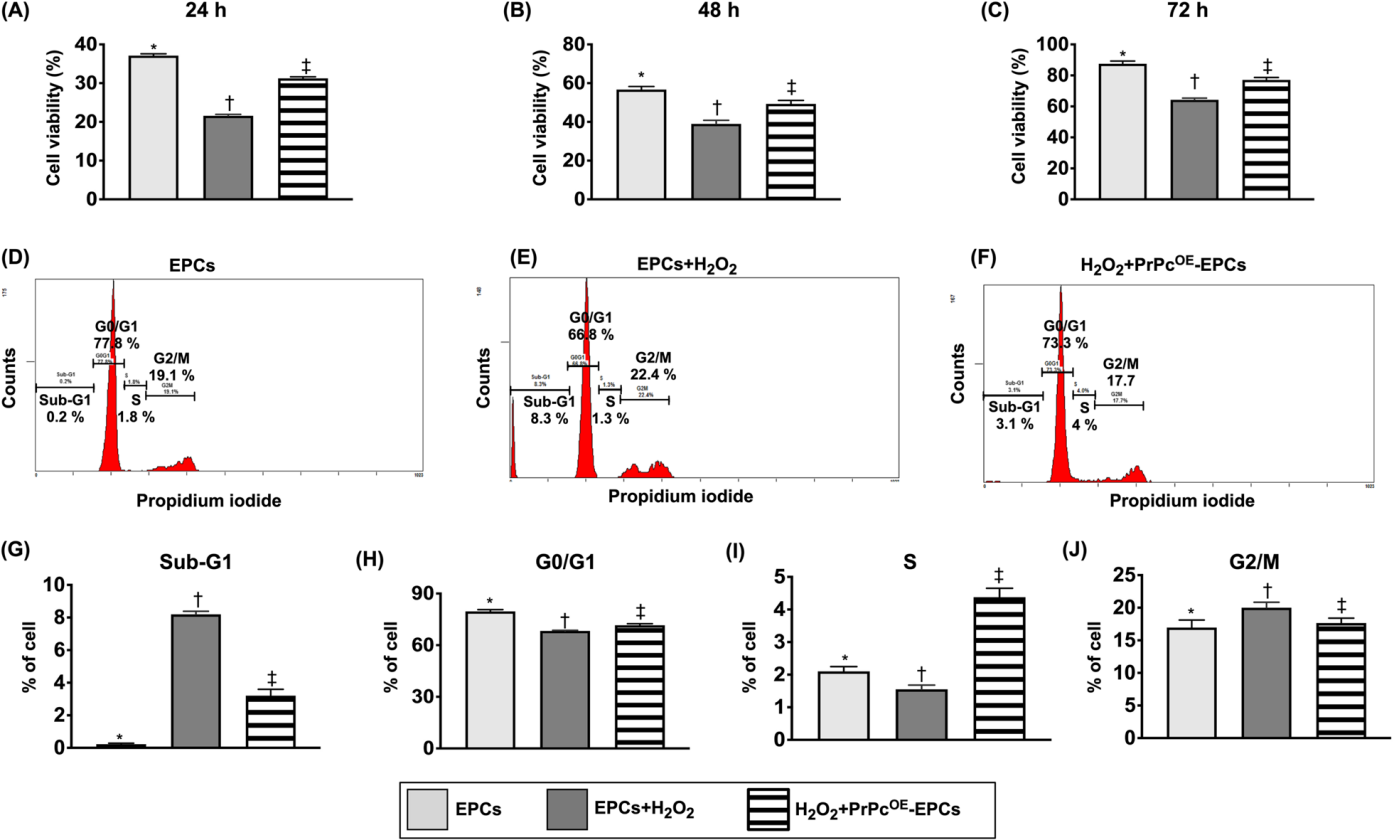
MTT assay for assessment of the impact of PrPc^OE^ on cell viability and cell cycle. **A** Cell viability at 24 h, * versus other groups with different symbols (†, ‡), *p* < 0.0001. **B** Cell viability at 48 h, * versus other groups with different symbols (†, ‡), *p* < 0.0001. **C** Cell viability at 72 h, * versus other groups with different symbols (†, ‡), *p* < 0.0001. **D**–**F)** Illustrating the flow cytometric analysis for determining the cell cycle activity in EPCs only **D**, EPCs + H_2_O_2_ **E** and H_2_O_2_ + PrPc^OE^-EPCs **F**, respectively. **G** Analytical result of the expression of Sub-G1 phase of cell cycle, * versus other groups with different symbols (†, ‡), *p* < 0.0001. **H** Analytical result of the expression of G0/G1 phase of cell cycle, * versus other groups with different symbols (†, ‡), *p* < 0.0001. **I** Analytical result of the expression of *S* phase of cell cycle, * versus other groups with different symbols (†, ‡), *p* < 0.0001. **J** Analytical result of the expression of G2/M phase of cell cycle, * versus †, *p* < 0.01. All statistical analyses were performed by one-way ANOVA, followed by Bonferroni multiple comparison post hoc test (*n* = 8 for each group). Data were expressed as mean ± SE. Symbols (*, †, ‡) indicate significance (at 0.05 level). EPCs = endothelial progenitor cells; H_2_O_2_ = hydrogen peroxide; and PrPc^OE^-EPCs = overexpression of cellular prion protein in EPCs

To evaluate the impact of PrPc^OE^ on inhibiting the expressions of total intracellular ROS and cellular apoptosis, the cells were categorized into E1–E3, as mentioned above, in Fig. 1. The results of flow cytometric analysis showed that the fluorescent intensities of total intracellular ROS and early and late apoptosis were significantly increased in E2 than in E1 that were significantly reversed in E3, suggesting PrPc^OE^-EPCs enhanced capacity of EPC resistance to ROS damage and apoptosis.

**Fig. 2:**
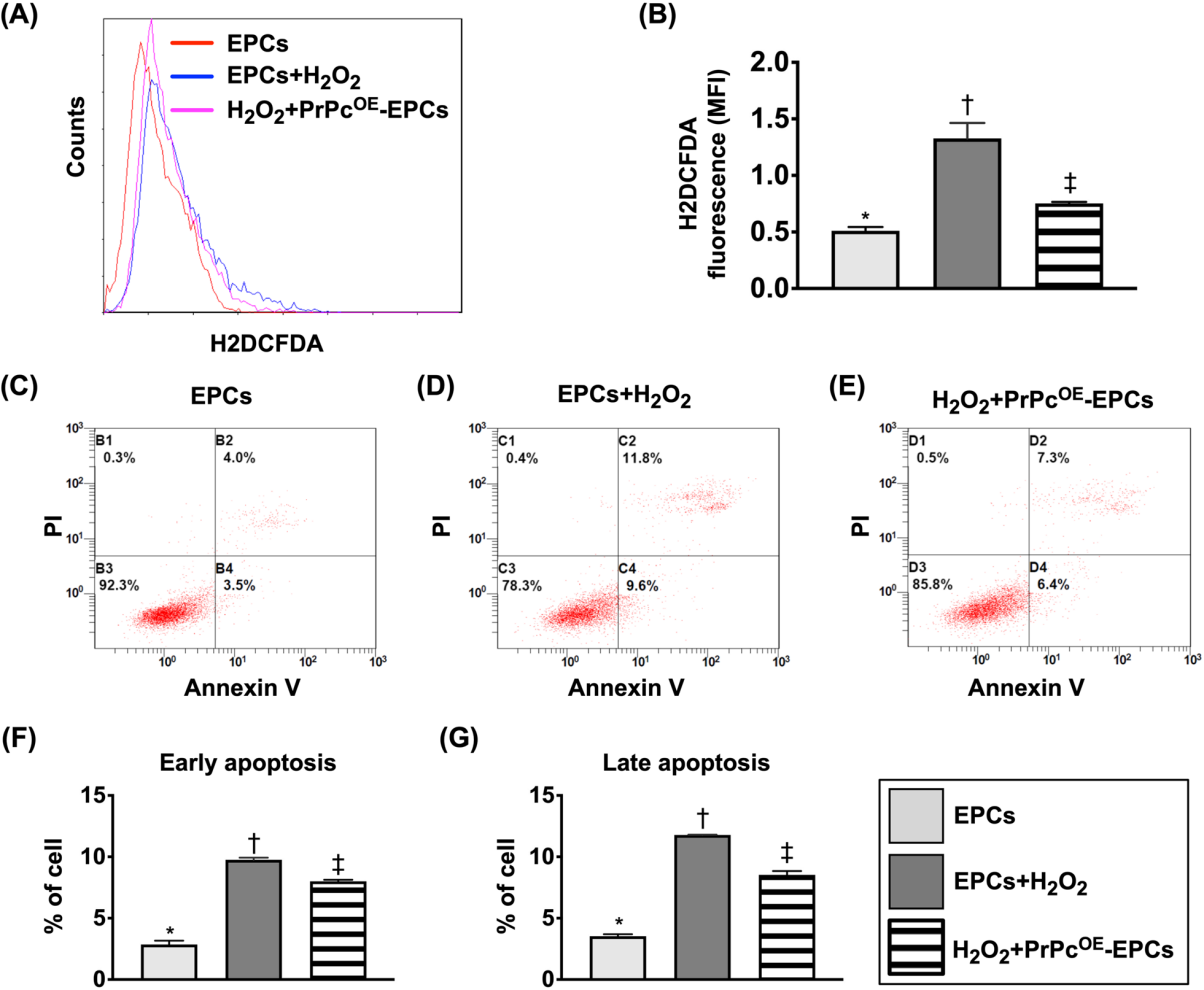
Impact of PrPc^OE^ on inhibiting the intracellular ROS and cell apoptosis. **A** Illustrating the flow cytometric analysis for identification of total intracellular reactive oxygen species (ROS). **B** Analytical result of mean fluorescent intensity (MFI) (i.e., stained by H_2_DCFDA dye) of total intracellular ROS, * versus other groups with different symbols (†, ‡), *p* < 0.0001. **C**–**E** Illustrating the flow cytometric analysis for determining the cellular apoptosis. **F** Flow cytometric result of early (AN-V +/PI −) apoptotic cells, * versus other group with different symbols (†, ‡), *p* < 0.0001. **G** Flow cytometric result of late (AN-V + /PI +) apoptotic cells, * versus other group with different symbols (†, ‡), *p* < 0.0001. All statistical analyses were performed by one-way ANOVA, followed by Bonferroni multiple comparison post hoc test (*n* = 8 for each group). Symbols (*, †, ‡) indicate significance (at 0.05 level). H_2_O_2_ = hydrogen peroxide; EPCs = endothelial progenitor cells; and PrPc^OE^-EPCs = overexpression of cellular prion protein in EPCs

**Fig. 3:**
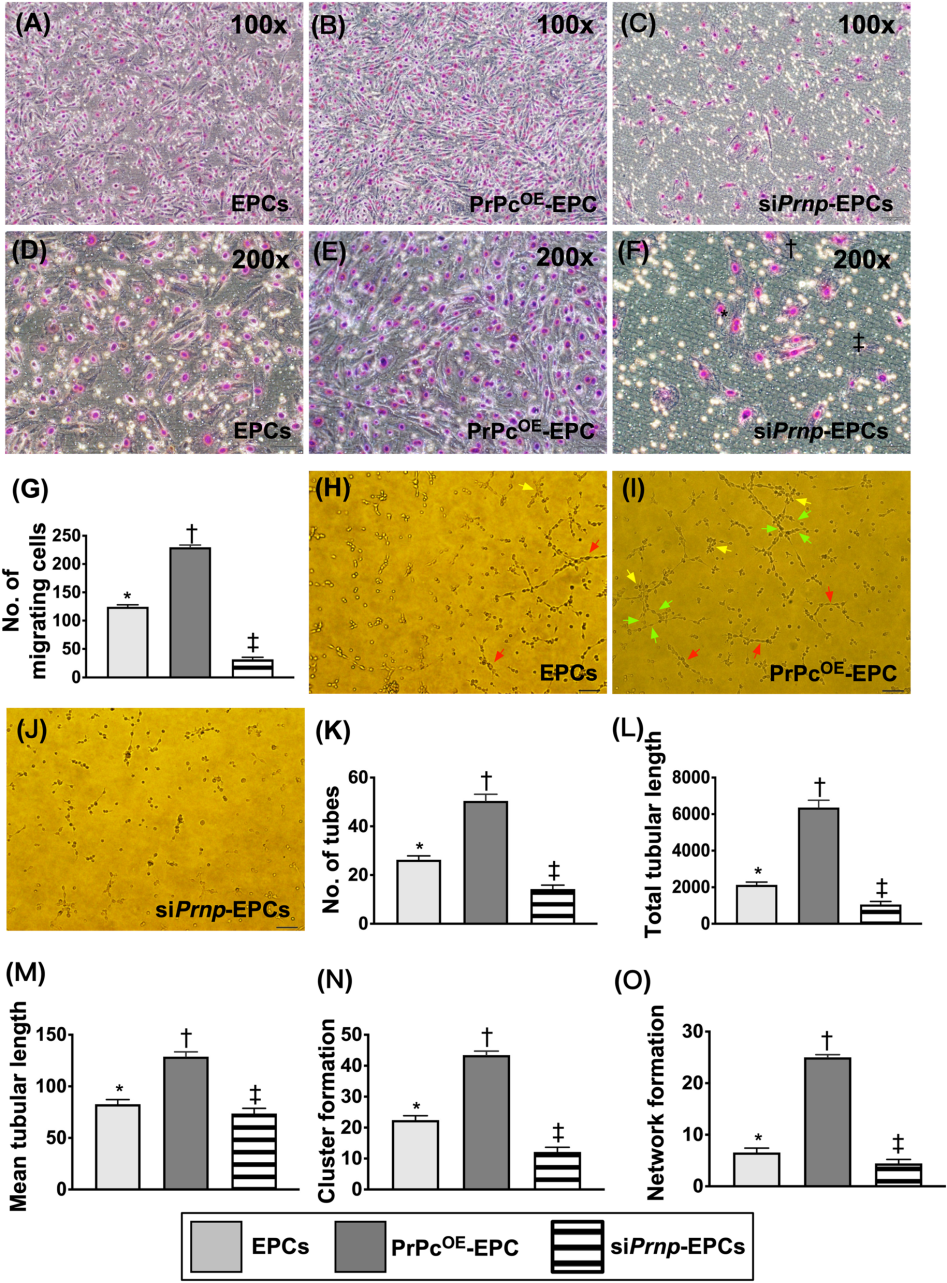
Impact of PrPc^OE^ on cell migratory and angiogenesis capacity.** A**–**F** illustrating the microscopic finding for identification of cell migratory capacity (i.e., by Migratory assay). **A**–**C**: indicated 100 × under microscopic finding; scale bars in right lower corner represent 100 µm. D to F: indicated 200 × under microscopic finding; scale bars in right lower corner represent 50 µm. **G** Analytical result of number of cell migration, * versus other group with different symbols (†, ‡, §), *p* < 0.0001. **H**–**J** Illustrating the morphological features (200×) of Matrigel assay for identification of angiogenesis in EPCs, PrPc^OE^-EPCs and si*Prnp*-EPCs, respectively. The parameters of angiogenesis, including: (1) tubular formation (red arrows), (2) cluster formation (yellow arrows) and (3) network formation (green color). **K** Analytical result of number of tubules, * versus other groups with different symbols (†, ‡), *p* < 0.0001. **L** Analytical result of total tubular length, * versus other groups with different symbols (†, ‡), *p* < 0.0001. **M** Analytical result of mean tubular length, * versus other groups with different symbols (†, ‡), *p* < 0.0001. **N** Analytical result of cluster formation, * versus other groups with different symbols (†, ‡), *p* < 0.0001. **O** Analytical result of network formation, * versus other groups with different symbols (†, ‡), *p* < 0.0001. Scale bar in right lower corner represents 50 µm. All statistical analyses were performed by one-way ANOVA, followed by Bonferroni multiple comparison post hoc test (*n* = 8 for each group). Symbols (*, †, ‡) indicate significance (at 0.05 level). EPCs = endothelial progenitor cells; PrPc^OE^-EPCs = overexpression of cellular prion protein in EPCs. si*Prnp*-EPCs = knockdown of cellular prion protein in EPCs

**Fig. 4:**
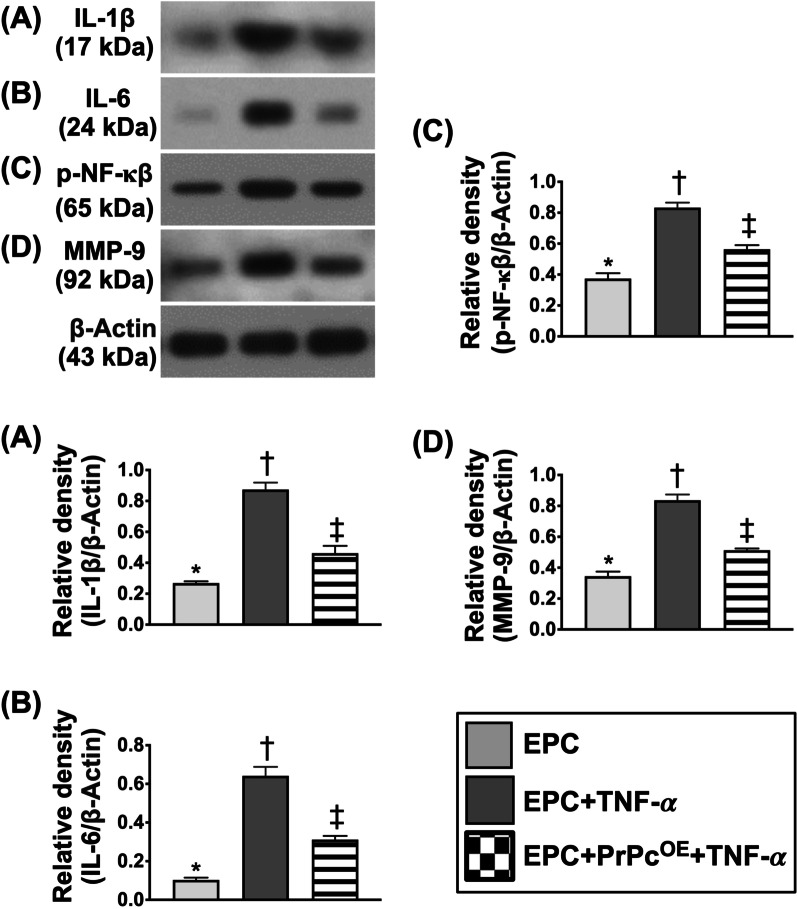
Impact of PrPc^OE^ on suppressing inflammation. **A** Protein expression of interleukin (IL-1ß), * versus other groups with different symbols (†, ‡), *p* < 0.001. **B** Protein expression of IL-6, * versus other groups with different symbols (†, ‡), *p* < 0.001. **C** Phosphorylated (p) nuclear factor (NF)-κB, * versus other groups with different symbols (†, ‡), *p* < 0.001. **D** Protein expression of matrix metalloproteinase (MMP-9), * versus other groups with different symbols (†, ‡), *p* < 0.0001. All statistical analyses were performed by one-way ANOVA, followed by Bonferroni multiple comparison post hoc test (*n* = 8 for each group). Symbols (*, †, ‡) indicate significance (at 0.05 level). PrPc^OE^ = overexpression of cellular prion protein; EPC = endothelial progenitor cells; and TNF-*α* = tumor necrosis factor alpha

**Fig. 5:**
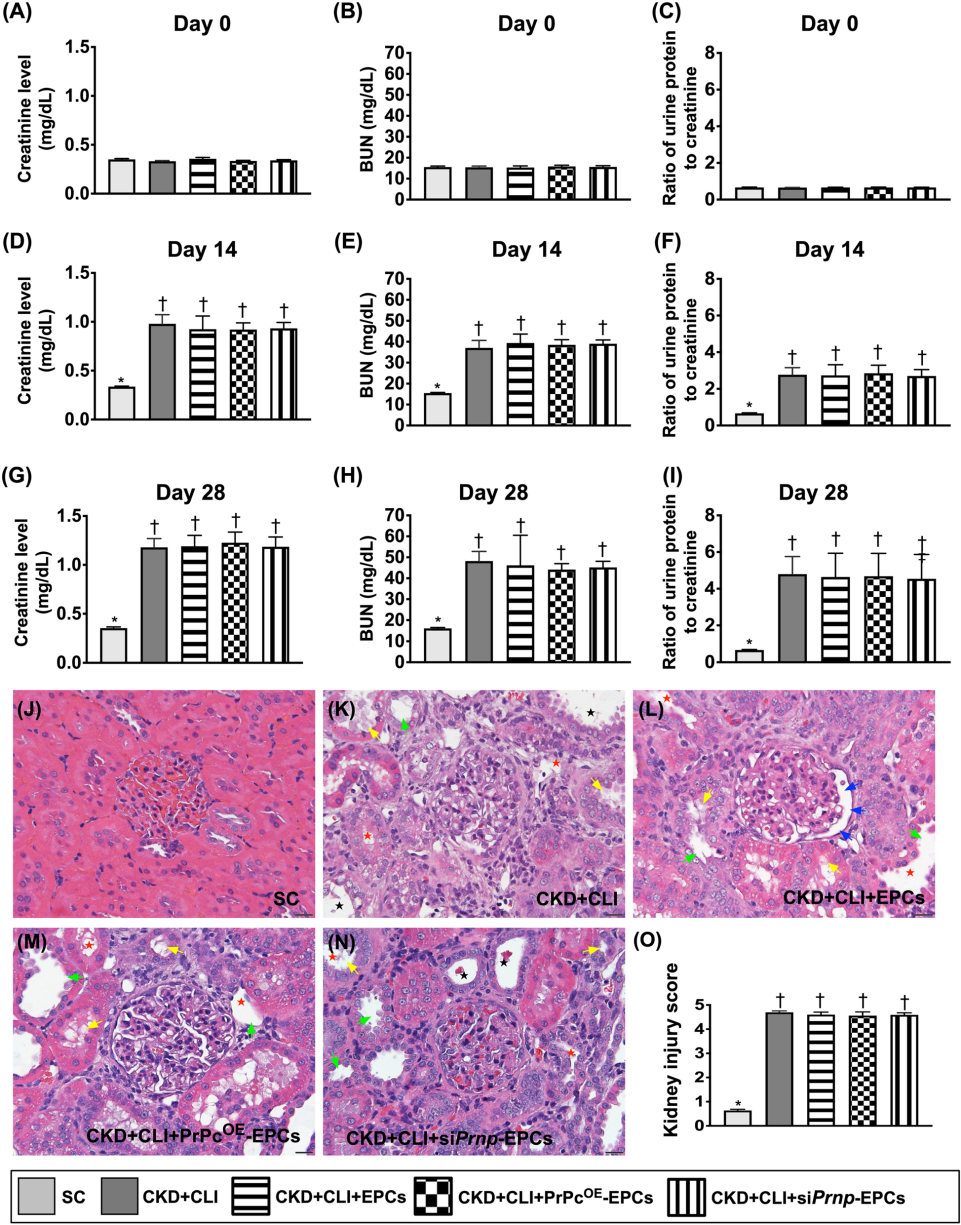
Time courses of circulating levels of blood urine nitrogen (BUN) and creatinine and ratio of urine protein to urine creatinine and kidney injury score by day 42 after CKD induction. **A** Circulating level of creatinine at day 0, *p* > 0.5. **B** Circulating level of BUN, at day 0, *p* > 0.5. **C** Ratio of urine protein to urine creatinine (RU_P/C_) at day 0, *p* > 0.5. **D** Circulating level of creatinine at day 14, * versus †, *p* < 0.001. **E** Circulating level of BUN, at day 14, *p* < 0.001. **F** Ratio of RU_P/C_ at day 14, * versus †, *p* < 0.001. **G** Circulating level of creatinine at day 28, * versus †, *p* < 0.0001. **H** Circulating level of BUN, at day 28, *p* < 0.0001. **I** Ratio of RU_P/C_ at day 28, * versus †, *p* < 0.001. **J**–**N)** Light microscopic findings (400×; H&E stain) showing significantly higher loss of brush border in renal tubules (yellow arrows), tubular necrosis (green arrows), tubular dilatation (red asterisk), protein cast formation (black asterisk), and dilatation of Bowman’s capsule (blue arrows) in CKD groups with and without treatment than in SC group. **G** Analytical result of kidney injury score, * versus †, *p* < 0.0001. *n* = 8 in each group. SC = sham-operated control; CKD = chronic kidney disease; CLI = critical limb ischemia; EPCs = endothelial progenitor cells; PrPc^OE^-EPCs = overexpression of cellular prion protein in EPCs; and si*Prnp*-EPCs = knockdown of cellular prion protein in EPCs

**Fig. 6:**
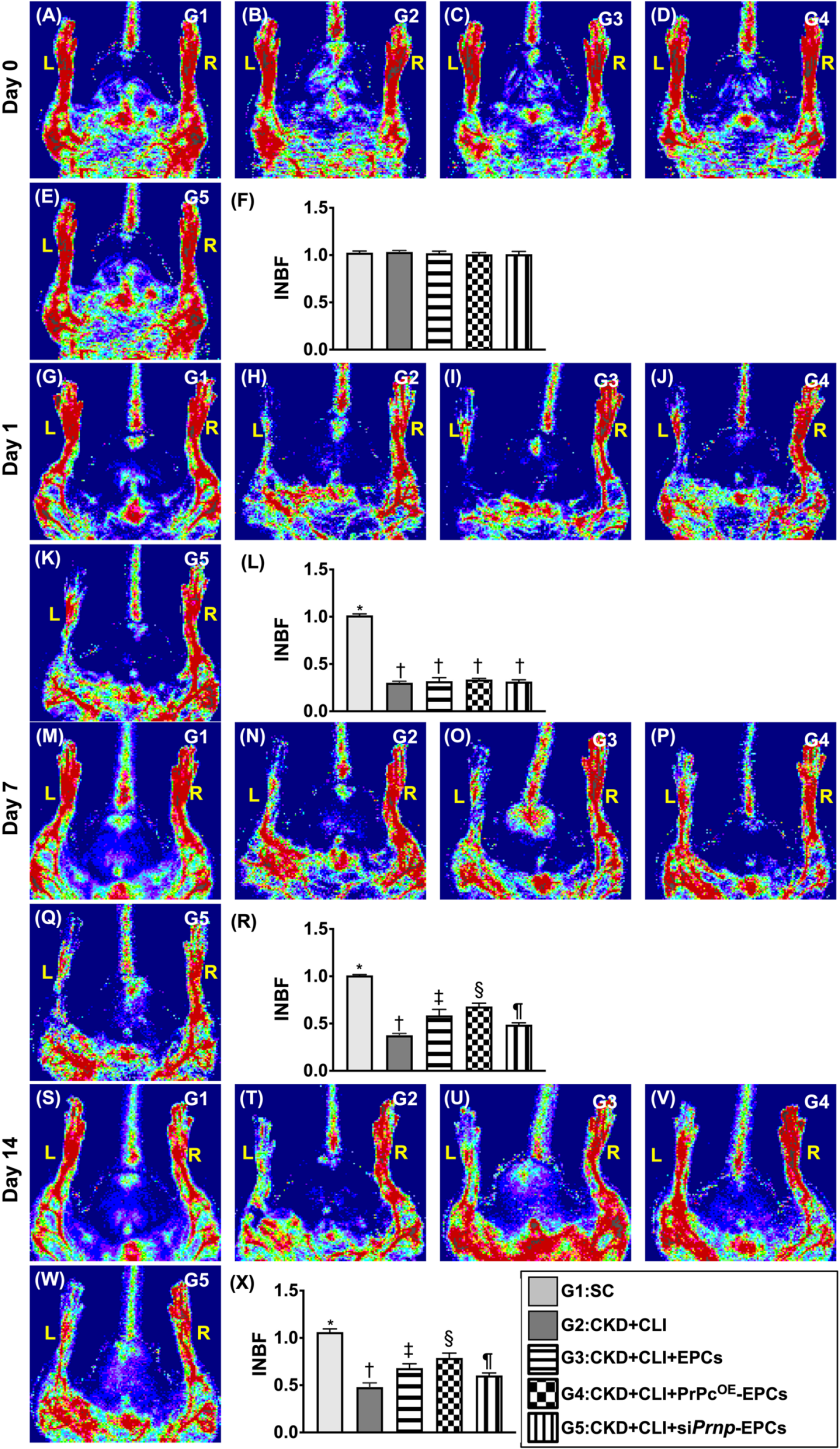
Ischemic to normal blood flow (INBF) ratio measured by laser Doppler scan at days 0, 1, 7 and 14 after left CLI induction. **A**–**E** Illustrating the laser Doppler finding of ratio of left hind limb (ischemia) to right hind limb (normal) blood flow (i.e., INBF) at day 0 prior to critical limb ischemia (CLI) procedure among the five groups. **F** Analytical result of ratio of INBF, *p* > 0.5. **G**–**K** Illustrating the laser Doppler finding of INBF at day 1 after CLI procedure among the five groups. **L** Analytical result of ratio of INBF, * versus †, *p* < 0.0001. **M**–**Q** Illustrating the laser Doppler finding of ratio of INBF at day 7 after CLI procedure among the five groups. **R** Analytical result of ratio of INBF, * versus other groups with different symbols (†, ‡, §, ¶), *p* < 0.0001. **S**–**W** Illustrating the laser Doppler finding of ratio of INBF at day 14 after CLI procedure among the FIVE groups. **X** Analytical result of ratio of INBF, * versus other groups with different symbols (†, ‡, §, ¶), *p* < 0.0001. Note that each rat was placed in a supine position and the tail in upward direction. Thus, the left side of animal indicated the left critical limb and the attenuated Doppler color, i.e., implicated a reduction in blood flow in left CLI area, was clearly observed. All statistical analyses were performed by one-way ANOVA, followed by Bonferroni multiple comparison post hoc test (*n* = 8 for each group). Symbols (*, †, ‡, §, ¶) indicate significance (at 0.05 level). L = left limb; R = limb; right SC = sham-operated control; CKD = chronic kidney disease; CLI = critical limb ischemia; EPCs = endothelial progenitor cells; PrPc^OE^-EPCs = overexpression of cellular prion protein in EPCs; and si*Prnp*-EPCs = knockdown of cellular prion protein in EPCs

**Fig. 7:**
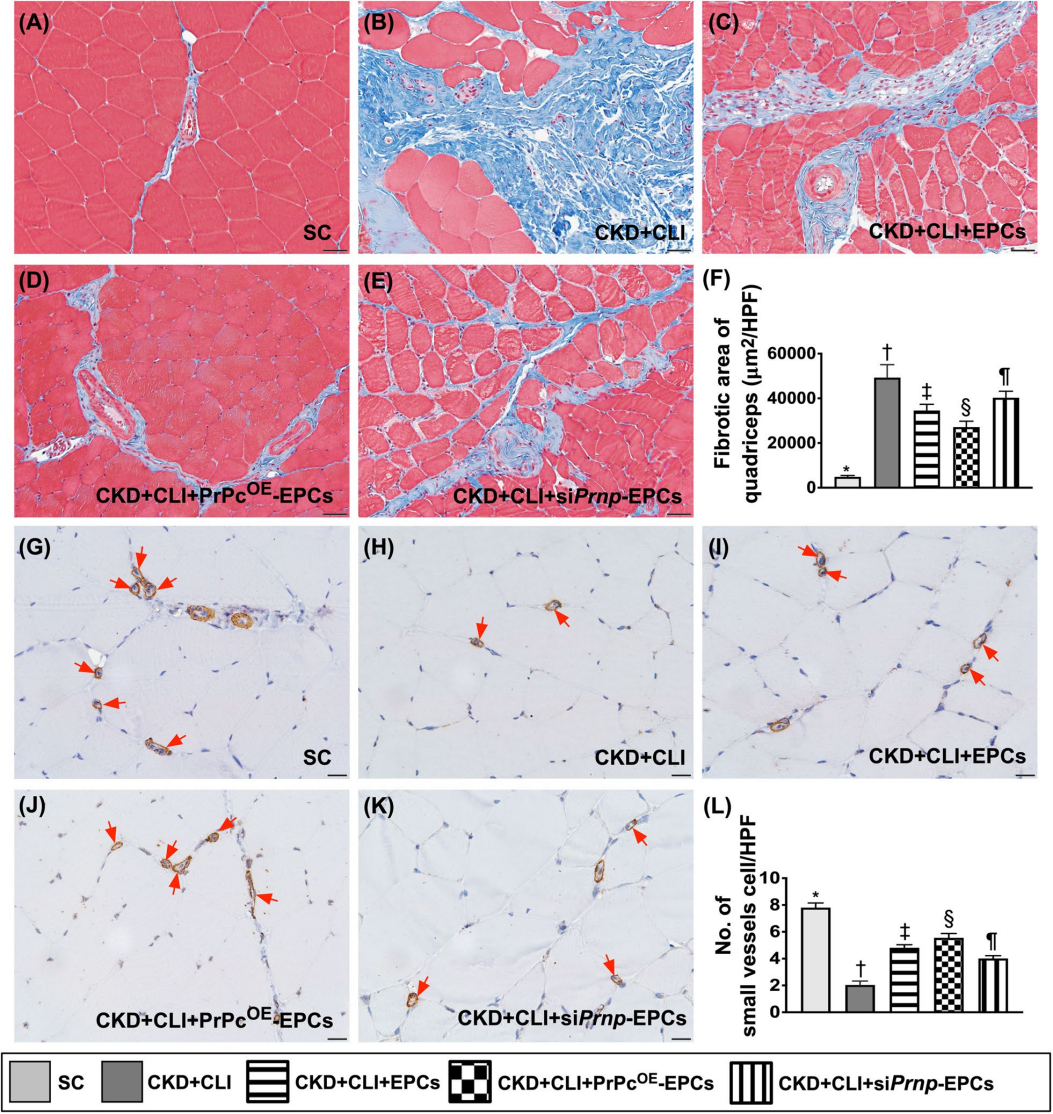
Fibrosis and small vessel density in quadriceps muscle by day 42 after CKD induction. **A**–**E** Illustrating the immunofluorescent (IHC) microscopic finding (200×) for identification of fibrotic area (blue color). **F** Analytical results of fibrotic area, * versus other groups with different symbols (†, ‡, §, ¶), *p* < 0.0001. Scale bar in right lower corner represents 50 µm. **G**–**K)** Illustrating the microscopic finding (400×) of alpha-smooth muscle actin (α-SMA) stain for identification of the expression of small vessels (i.e., diameter ≤ 25 μM) (red arrows). **L** Analytical result of number of small vessels, * versus other groups with different symbols (†, ‡, §, ¶), *p* < 0.0001. Scale bar in right lower corner represents 20 µm. All statistical analyses were performed by one-way ANOVA, followed by Bonferroni multiple comparison post hoc test (*n* = 8 for each group). Symbols (*, †, ‡, §, ¶) indicate significance (at 0.05 level). HPF = high-power field; SC = sham-operated control; CKD = chronic kidney disease; CLI = critical limb ischemia; EPCs = endothelial progenitor cells; PrPc^OE^-EPCs = overexpression of cellular prion protein in EPCs; and si*Prnp*-EPCs = knockdown of cellular prion protein in EPCs

**Fig. 8:**
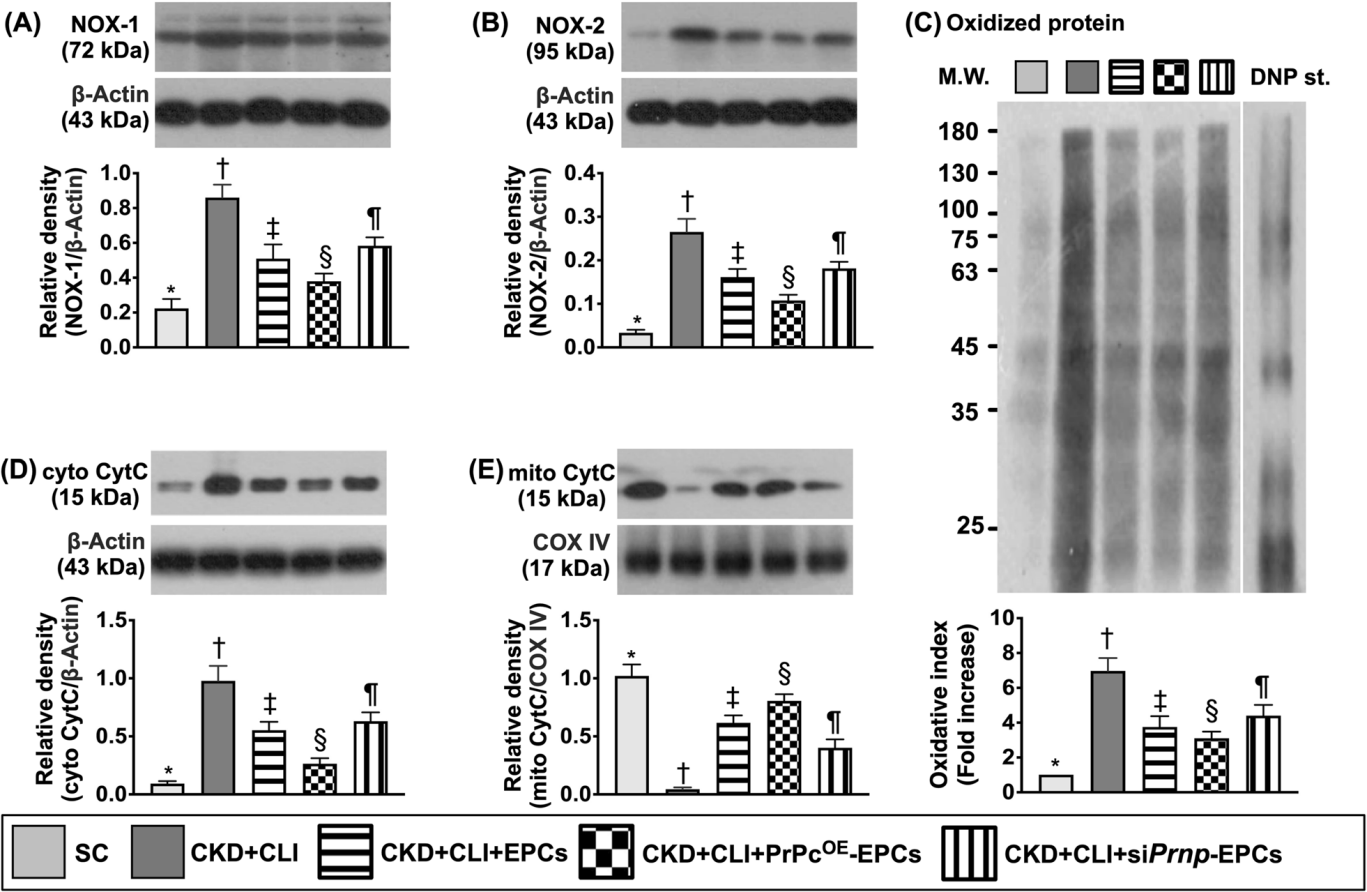
Protein expressions of oxidative stress and mitochondrial damaged biomarkers in ischemic quadriceps muscle by day 42 after CKD induction. **A** Protein expression of NOX-1, * versus other groups with different symbols (†, ‡, §, ¶), *p* < 0.001. **B** Protein expression of NOX-2, * versus other groups with different symbols (†, ‡, §, ¶), *p* < 0.001. **C** Illustrating the Western blot band, i.e., by using the Oxyblot Oxidized Protein Detection Kit for detection; analytical result of oxidized protein expression, * versus other groups with different symbols (†, ‡, §, ¶), *p* < 0.0001 (*Note*: the left and right lanes shown on the upper panel represent protein molecular weight marker and control oxidized molecular protein standard, respectively). M.W. = molecular weight; DNP = 1–3 dinitrophenylhydrazone. **D** Protein expression of cytosolic cytochrome C (Cyt-cyto-C), * versus other groups with different symbols (†, ‡, §, ¶), *p* < 0.0001. **E** Protein expression of mitochondrial cytochrome C (Mit-cyto-C), * versus other groups with different symbols (†, ‡, §, ¶), *p* < 0.0001. All statistical analyses were performed by one-way ANOVA, followed by Bonferroni multiple comparison post hoc test (*n* = 6 for each group). Symbols [(*, †, ‡, §, ¶) indicate significance (at 0.05 level). SC = sham-operated control; CKD = chronic kidney disease; CLI = critical limb ischemia; EPCs = endothelial progenitor cells; PrPc^OE^-EPCs = overexpression of cellular prion protein in EPCs; and si*Prnp*-EPCs = knockdown of cellular prion protein in EPCs

**Fig. 9:**
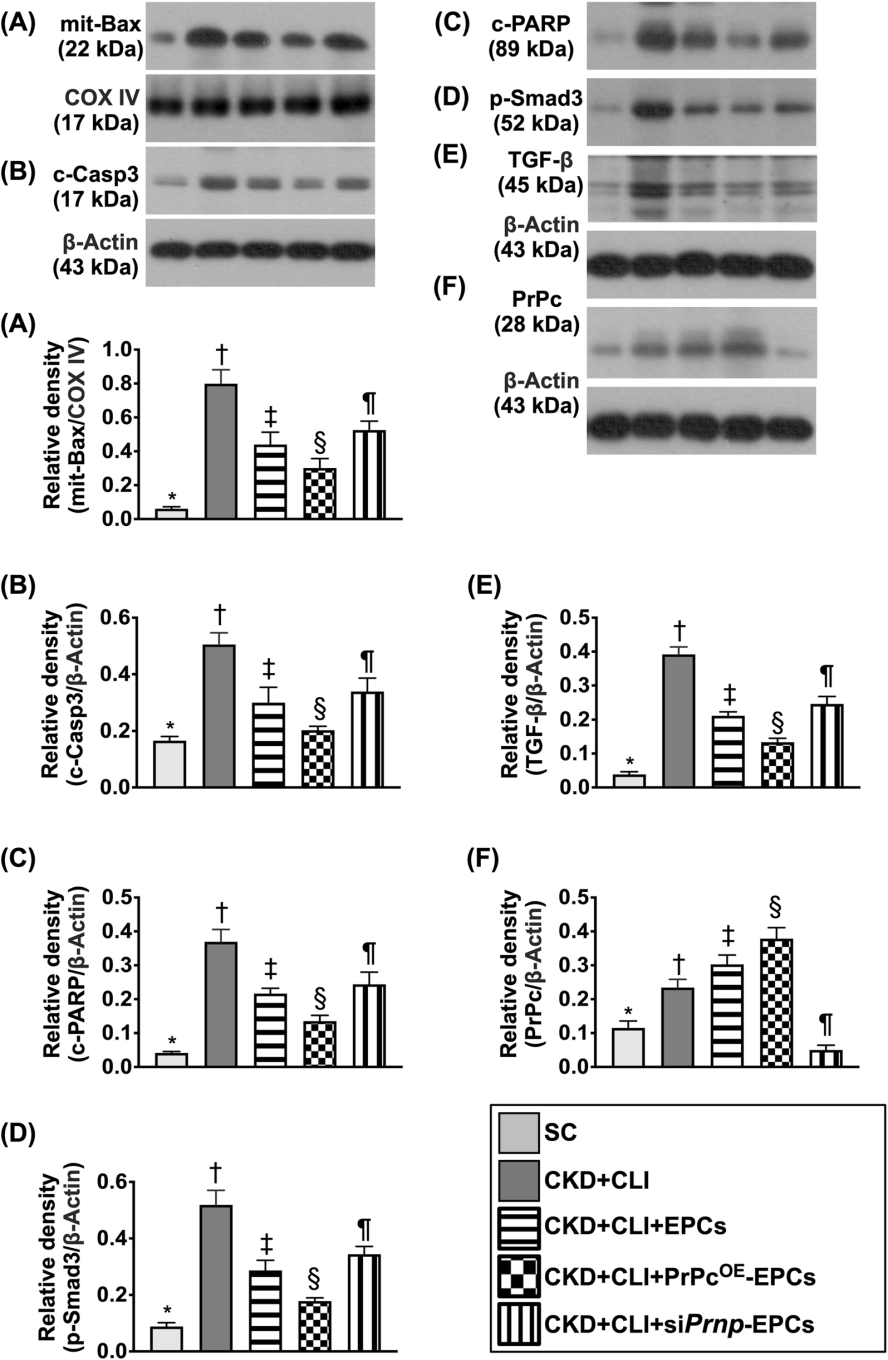
The protein expressions of apoptotic and fibrotic biomarkers and PrPc in ischemic quadriceps muscle by day 42 after CKD induction. **A** Protein expression of mitochondrial Bax (Mit-Bax), * versus other groups with different symbols (†, ‡, §, ¶), *p* < 0.0001. **B** Protein expression of cleaved caspase 3 (c-Casp3), * versus other groups with different symbols (†, ‡, §, ¶), *p* < 0.0001. **C** Protein expression of cleaved poly (ADP-ribose) polymerase (c-PARP), * versus other groups with different symbols (†, ‡, §, ¶), *p* < 0.0001. **D** Protein expression of phosphorylated (p)-Smad3, * versus other groups with different symbols (†, ‡, §, ¶), *p* < 0.0001. **E** Protein expression of transforming growth factor (TGF)-ß, * versus other groups with different symbols (†, ‡, §, ¶), *p* < 0.0001. **F** Protein expression of cellular prion protein (PrPc), * versus other groups with different symbols (†, ‡, §, ¶), *p* < 0.0001. All statistical analyses were performed by one-way ANOVA, followed by Bonferroni multiple comparison post hoc test (*n* = 6 for each group). Symbols [(*, †, ‡, §, ¶) indicate significance (at 0.05 level). SC = sham-operated control; CKD = chronic kidney disease; CLI = critical limb ischemia; EPCs = endothelial progenitor cells; PrPc^OE^-EPCs = overexpression of cellular prion protein in EPCs; and si*Prnp*-EPCs = knockdown of cellular prion protein in EPCs

**Fig. 10:**
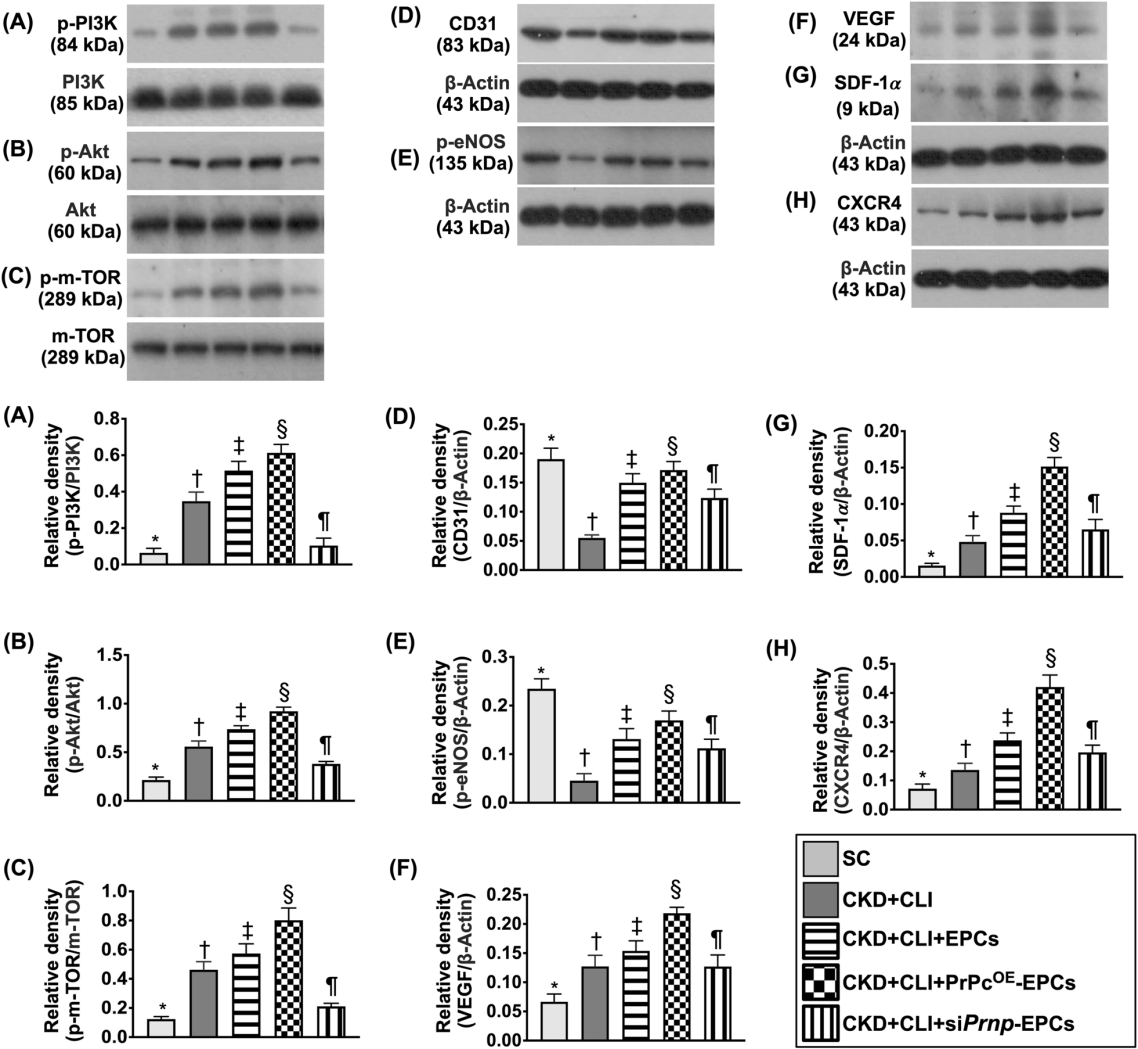
The protein expressions of cell-stress/cell-proliferation signaling and angiogenesis in ischemic quadriceps muscle by day 42 after CKD induction. **A** Protein expression of phosphorylated (p)-PI3K, * versus other groups with different symbols (†, ‡, §, ¶), *p* < 0.0001. **B** Protein expression of p-Akt, * versus other groups with different symbols (†, ‡, §, ¶), *p* < 0.0001. **C** Protein expression of p-m-TOR, * versus other groups with different symbols (†, ‡, §, ¶), *p* < 0.0001. **D** Protein expression of CD31, * versus other groups with different symbols (†, ‡, §, ¶), *p* < 0.0001. **E** Protein expression of phosphorylated endothelial nitric oxide synthase (*p*-eNOS), * versus other groups with different symbols (†, ‡, §, ¶), *p* < 0.0001. **F** Protein expression of vascular endothelial growth factor (VEGF), * versus other groups with different symbols (†, ‡, §, ¶), *p* < 0.0001. **G** Protein expression of stromal cell-derived factor (SDF)-1α, * versus other groups with different symbols (†, ‡, §, ¶), *p* < 0.0001. **H** Protein expression of CXCR4, * versus other groups with different symbols (†, ‡, §, ¶), *p* < 0.0001. All statistical analyses were performed by one-way ANOVA, followed by Bonferroni multiple comparison post hoc test (*n* = 6 for each group). Symbols [(*, †, ‡, §, ¶) indicate significance (at 0.05 level). SC = sham-operated control; CKD = chronic kidney disease; CLI = critical limb ischemia; EPCs = endothelial progenitor cells; PrPc^OE^-EPCs = overexpression of cellular prion protein in EPCs; and si*Prnp*-EPCs = knockdown of cellular prion protein in EPCs

### Impact of PrPc^OE^ on cell migratory and angiogenesis capacity (Figure 3)

To verify whether PrPc^OE^-EPCs in rat derived EPCs would enhance the capacity of cell migration and angiogenesis (i.e., by Matrigel assay), the cells were categorized into EPCs only, PrPc^OE^-EPCs and si*Prnp* in EPCs. As we expected, the capacities of cell migration and angiogenesis were significantly lower in si*Prnp* in EPCs than in EPCs only that were significantly reversed in PrPc^OE^-EPCs, indicating that PrPc served as an essential role for migration and angiogenesis of EPCs.

### Impact of PrPc^OE^ on suppressing inflammatory reaction (Figure 4)

To clarify whether PrPc^OE^ would suppress the inflammatory stimulation, the circulatory EPCs derived from healthy rats were categorized into EPCs only, EPCs + TNF-*α* (10 ng) treated for 4 days and PrPc^OE^-EPCs + TNF-*α* (10 ng) treated for 4 days, respectively. The Western blot analysis demonstrated that the protein expressions of IL-1ß, IL-6, *p*-NF-κB and MMP-9, four indicators of inflammation, were significantly increased in EPCs + TNF-*α* as compared with EPCs only that were significantly reversed in PrPc^OE^-EPCs + TNF-α, implying that PrPc^OE^-EPCs would ameliorate inflammation.

### Time courses of circulating levels of creatinine and BUN, and kidney injury score by day 42 after CKD induction (Figure 5)

To verify the successful procedure of CKD induction, the time courses of circulatory levels of BUN and creatinine and the ratio of urine protein to urine creatinine (RUc/*p*) were prospectively measured by collecting the peripheral blood and urine samplings, respectively. The result showed that the baseline levels of these three parameters did not differ among the five groups, i.e., group 1 (sham-operated control), group 2 (CKD + CLI), group 3 (CKD + CLI + EPCs), group 4 (CKD + CLI + PrPc^OE^-EPCs) and group 5 (CKD + CLI + si*Prnp*-EPCs). However, by days 14 and 28 after CKD induction, these parameters were significantly increased in groups 2–5 than in those of group 1, but they did not differ among the groups 2–5, implying that our CKD animal model was successfully created.

By day 42 after CKD induction, we collected the kidney specimen for kidney injury analysis. The result showed that the kidney injury score exhibited an identical pattern of day-28 circulating level of creatinine among the groups.

### Ischemic to normal blood flow (INBF) ratio measured by laser Doppler scan at days 0, 1, 7 and 14 after left CLI induction (Figure 6)

As we expected, by day 28 after CKD induction and prior to CLI induction, laser Doppler examination revealed that the INBF ratio did not differ among the groups. However, by day 1 after CLI induction, laser Doppler examination demonstrated a significantly higher INBF ratio in group 1 than in groups 2–5, but this parameter did not show obvious difference among the groups 2–5. By days 7 and 14 after induction of CLI, the ratio of INBF was highest in group 1, lowest in group 2, significantly lower in group 5 than in groups 3 and 4 and significantly lower in group 3 than in group 4.

### Histopathological findings of quadriceps muscle by day 42 after CKD induction (Figure 7)

The Masson’s trichrome stain identified that the fibrosis of the ischemic area was highest in group 2, lowest in group 1, significantly higher in group 5 than in groups 3 and 4 and significantly higher in group 3 than in group 4. On the other hand, the number of small vessel (i.e., ≤ 25 μM), an indicator of angiogenesis/vasculogenesis, displayed an opposite pattern of fibrosis among the groups.

### The protein expressions of oxidative stress and mitochondrial damage biomarkers in ischemic quadriceps muscle by day 42 after CKD induction (Figure 8)

The protein expressions of NOX-1, NOX-2 and oxidized protein, three indicators of oxidative stress, were highest in group 2, lowest in group 1, significantly lower in group 4 than in groups 3 and 5 and significantly lower in group 3 than in group 5. Additionally, the protein expression of cytosolic cytochrome C, an indicator of mitochondrial damage, exhibited an identical pattern of oxidative stress among the groups. On the other hand, the protein expression of mitochondrial cytochrome C, an indicator of mitochondrial integrity, displayed an opposite pattern of oxidative stress. Our findings implicated that PrPc^OE^-EPCs would be more resistant to oxidative stress damage in ischemic region.

### The protein expressions of apoptotic and fibrotic biomarkers and PrPc in ischemic quadriceps muscle by day 42 after CKD induction (Figure 9)

To delineate whether PrPc^OE^-EPCs treatment would better attenuate the protein levels of apoptosis and fibrosis, the Western blot analysis was utilized in the present study. Our results demonstrated that the protein expressions of mitochondrial Bax, cleaved caspase 3 and cleaved PARP, three indicators of apoptosis, and the protein expressions of Smad3 and TGF-ß, two indices of fibrosis, were highest in group 2, lowest in group 1, significantly lower in group 4 than in groups 3 and 5 and significantly lower in group 3 than in group 5. The protein expression of PrPc was lowest in groups 2 and 5, highest in group 4 and significantly higher in group 1 than in group 3, suggesting that this parameter would be suppressed in ischemic tissue.

### The protein expressions of cell-stress/cell-proliferation signaling and angiogenesis in ischemic quadriceps muscle by day 42 after CKD induction (Figure 10)

To clarify the cell-stress/cell-proliferation signaling would be activated to protect against the ischemic stimulation, Western blot analysis was utilized again in the present study. As we expected, the protein levels of p-PI3K, p-Akt and p-m-TOR, three cell-stress/cell-proliferation signaling biomarkers, were lowest in group 1, highest in group 4, significantly lower in groups 1 and 5 than in group 3 and significantly lower in group 1 than in group 5.

The protein expressions of CD31 and *p*-eNOS, two endothelial cell surface/angiogenesis biomarkers, were highest in group 1, lowest in group 2, significantly lower in group 5 than in groups 3 and 4 and significantly lower in group 3 than in group 4. Additionally, the protein expressions of SDF-1α, CXCR4 and VEGF, three indices of angiogenesis, were significantly and progressively increased from groups 1–4 that were reversed in group 5, implicating an intrinsic response to ischemic stress that was upregulated by EPC treatment and further upregulated by PrPc^OE^-EPCs treatment.

### Cellular levels of fibrosis and podocyte components and kidney injury markers in kidney parenchyma by day 42 after CKD induction (Additional files 1 and 2: Figs. S1, S2)

The final aim of this study was to investigate whether relatively lower dose of EPCs or PrPc^OE^-EPCs treatment would protect the kidney parenchyma against CKD injury, especially when intravenous route of cell administration was conducted. The result demonstrated that the fibrotic area was significantly higher in groups 2–5 than in group 1, and significantly higher in groups 2, 3 and 5 than in group 4, but it showed no significant difference among the groups 2, 3 and 5 (Additional file 1: Fig. S1). Additionally, the IF microscopic finding demonstrated that the expression of KIM-1, an indicator of kidney damage marker predominantly distributed in renal tubules, exhibited an identical pattern of fibrosis among the five groups (Additional file 1: Fig. S1).

Furthermore, the IF microscopic findings demonstrated that the expressions of ZO-1 and synaptopodin, two indicators of podocyte components, predominantly localized in the glomeruli, exhibited an opposite pattern of fibrosis among the five groups (Additional file 2: Fig. S2). Our findings implicated that intravenous administration of a low dose of EPCs might not offer any additional benefit significant for protecting the kidney architectural integrity against the CKD damage. However, this phenomenon might be reversed by PrPc^OE^-EPCs administration.

## Discussion

Our study which investigated the therapeutic impact of PrPc^OE^-EPCs on setting of CLI coexisting with CKD revealed several striking implications. First, based on our results, we successfully created a CLI model in CKD rodent that just mimicked the clinical setting of CKD patients, followed by development of CLI which has been clearly identified to be associated with unacceptably high risks of poor prognostic outcomes [13–16]. Second, a therapy with PrPc^OE^-EPCs (i.e., defined as rejuvenation of EPCs) was superior to EPCs only for restoring the blood flow in ischemic area, salvaging the CLI and improving the outcomes. Third, the result of this study further demonstrated that the PI3K/Akt/m-TOR signaling pathway played a cardinal role for salvaging the CLI in rodent.

Although abundant data have proved that EPCs therapy effectively restored the blood flow in CLI area [34–36] or preserved the residual renal function in CKD [34] in rodent, the impact of this kind of stem cell therapy for salvaging the CLI in preexisting CKD has seldom been reported in experimental studies. The most important finding in the present study was that as compared with the CKD-CLI animals, the blood flow in the CLI was significantly improved in CKD-CLI animals after receiving the EPCs therapy and further significantly improved in that of CKD-CLI animals after receiving PrPc^OE^-EPCs, i.e., “rejuvenated EPCs” therapy. Our finding, in addition to extending the findings from previous studies [34–36], implicated that PrPc^OE^-EPCs could be superior to EPCs only in salvaging the CLI.

An essential finding not only in the in vitro but also in the in vivo studies was that as compared with EPCs only, the cell proliferation and cell cycle were remarkably increased in PrPc^OE^-EPCs. On the other hand, the oxidative stress, inflammatory reaction and mitochondrial damage were remarkably suppressed by PrPc^OE^-EPCs than by EPCs only. Interestingly, our recent studies [27, 29] have also demonstrated that PrPc^OE^-EPCs possessed the capacity of cell proliferation [29] and upregulation of PrPc-inhibited oxidative stress [27]. In this way, our finding in the present study was comparable with the findings in our recent studies [27, 29]. Additionally, our in vitro and in vivo studies further demonstrated that the angiogenesis capacity of PrPc^OE^-EPCs was superior to EPCs only. Our findings, i.e., including cell proliferation and angiogenesis, implicated that PrPc^OE^-EPCs could play a fundamental role of regenerative medicine for, at least in part, salvaging the CLI.

It was well known that ischemia always induced fibrosis and apoptosis in tissues and organs [27, 29–32]. A principal finding in the present study was that the molecular–cellular levels of fibrosis and the protein level of apoptosis were remarkably increased in CLI as compared to the control group. Accordingly, our finding coincided with the findings of previous studies [27, 29–32]. Importantly, we found that EPCs therapy remarkably and PrPc^OE^-EPCs therapy even more remarkably suppressed these molecular–cellular perturbations. These findings could partially explain why these EPCs/PrPc^OE^-EPCs therapies could effectively restore the blood flow and salvage the CLI.

Our recent study demonstrated that combined ADMSCs and valsartan or combined ADMSCs and melatonin therapy effectively entreasured the residual renal function in CKD rat mainly through upregulating the PrPc involvement in promoting the PI3K/Akt/m-TOR signaling and cell proliferation [27]. Intriguingly, the result of our in vitro study clearly identified that PrPc^OE^ in EPCs enhanced these cells proliferation and angiogenesis that were substantially suppressed by knockdown-PrPc in EPCs. Furthermore, the result of our in vivo study revealed that PrPc^OE^-EPCs augmented the expressions of PI3K/Akt/m-TOR biomarkers in the quadriceps muscle that were also notably weakened by knockdown-PrPc in EPCs. In this way, our findings, in addition to corroborating with the findings of a recent report [27], highlight that PI3K/Akt/m-TOR signaling participates in tissue regeneration, resulting in salvaging the CLI in rodent.

Our previous study has demonstrated that intrarenal arterial administration of autologous EPCs effectively preserved the renal function in setting of CKD rat [32]. However, in the present study when carefully scrutinized the molecular–cellular levels of the parameters (i.e., including podocyte components, KIM-1, kidney injury score) and the results of circulating levels of BUN and creatinine as well as the ratio of urine protein to urine creatinine, we did not find the evidence that EPCs therapy could significantly improve the rat renal function and the integrity of kidney architecture. Perhaps, this could be reasonably attributed to the following reasons. Frist, the dosage of the EPCs was relatively low (i.e., only 0.5 × 10^6^ cells) for treatment of CKD. Second, the route for administration of EPCs was transvenous, resulting in majority of the cells that were trapped in the lung parenchyma, especially in a chronic phase of CKD. Therefore, only an inadequate amount of the EPCs entered to the kidney to participate in the rescue of the renal function in CKD rat. These findings, therefore, provide an illuminating insight when we utilize the stem cell therapy, the dosage and administrative route of the utilized stem cells should be judiciously considered. Interestingly, our finding demonstrated that PrPc^OE^-EPCs treatment could improve the shortcomings of EPCs in this way.

### Study limitation

Our study has limitations. The study period of CLI was relatively short, i.e., only 14 days from CLI induction to euthanize the animals for individual study. Thus, the long-term impact of EPCs/PrPc^OE^-EPCs therapy on maintaining the blood flow in CLI remains uncertain. Second, we did not test the impact of two doses versus one dose of EPCs or PrPc^OE^-EPCs on improving the outcomes of the CLI in rodent. Third, we also did not test whether the stepwise-increased dosage of EPCs or PrPc^OE^-EPCs would offer an additional benefit on restoring the blood flow in CLI area.

In conclusion, the results of the present study demonstrated that as compared with the EPCs only, PrPc^OE^-EPCs offer additional benefits on restoring the blood flow in ischemic area and salvaging the CLI in rodent.

## Supplementary Information

**Additional file 1**: **Fig. S1**. Cellular levels of fibrosis and kidney injury biomarker in kidney parenchyma by day 42 after CKD induction. **A**–**E** illustrating the microscopic finding (200×) of Masson’s trichrome stain for identification of fibrosis in kidney parenchyma (blue color). **F** Analytical result of fibrotic area, * versus other groups with different symbols (†, ‡), *p* < 0.0001. *p* < 0.0001. All scale bars in right lower corner represent 50 µm. **G**–**K** Illustrating immunofluorescent (IF) microscopic finding (400×) for identification of kidney injury molecule (KIM-1) (green color). **L** Analytical result of expression of KIM-1, * vs. other groups with different symbols (†, ‡), *p* < 0.0001. *p* < 0.0001. Scale bars in right lower corner represent 20 µm. All statistical analyses were performed by one-way ANOVA, followed by Bonferroni multiple comparison post hoc test (*n* = 8 for each group). Symbols [(*, †, ‡) indicate significance (at 0.05 level). HPF = high-power field; SC = sham-operated control; CKD = chronic kidney disease; CLI = critical limb ischemia; EPCs = endothelial progenitor cells; PrPcOE-EPCs = overexpression of cellular prion protein in EPCs; siPrnp-EPCs = knockdown of cellular prion protein in EPCs

**Additional file 2**: **Fig. S2**. Cellular expressions of podocyte components in glomeruli by day 42 after CKD induction. **A**–**E** Illustrating the immunofluorescent (IF) microscopic finding (400×) for identification of ZO-1 in glomeruli (green color). **F** Analytical result of expression of ZO-1, * versus other groups with different symbols (†, ‡), *p* < 0.0001. **G**–**K** Illustrating IF microscopic finding (400×) for identification of synaptopodin (green color). **L** Analytical result of expression of synaptopodin, * vs. other groups with different symbols (†, ‡), *p* < 0.0001. *p* < 0.0001. Scale bars in right lower corner represent 20 µm. All statistical analyses were performed by one-way ANOVA, followed by Bonferroni multiple comparison post hoc test (*n* = 8 for each group). Symbols [(*, †, ‡) indicate significance (at 0.05 level). SC = sham-operated control; CKD = chronic kidney disease; CLI = critical limb ischemia; EPCs = endothelial progenitor cells; PrPcOE-EPCs = overexpression of cellular prion protein in EPCs; siPrnp-EPCs = knockdown of cellular prion protein in EPCs

## Data Availability

The data that support the findings of this study are available from the corresponding authors upon reasonable request.
