# Supplementary figures and images for: Rejuvenated endothelial progenitor cells through overexpression of cellular prion protein effectively salvaged the critical limb ischemia in rats with preexisting chronic kidney disease
Source: Stem Cell Res Ther. 2022 Sep 2;13:447. doi: 10.1186/s13287-022-03119-0 (PMC9440498; doi:10.1186/s13287-022-03119-0)

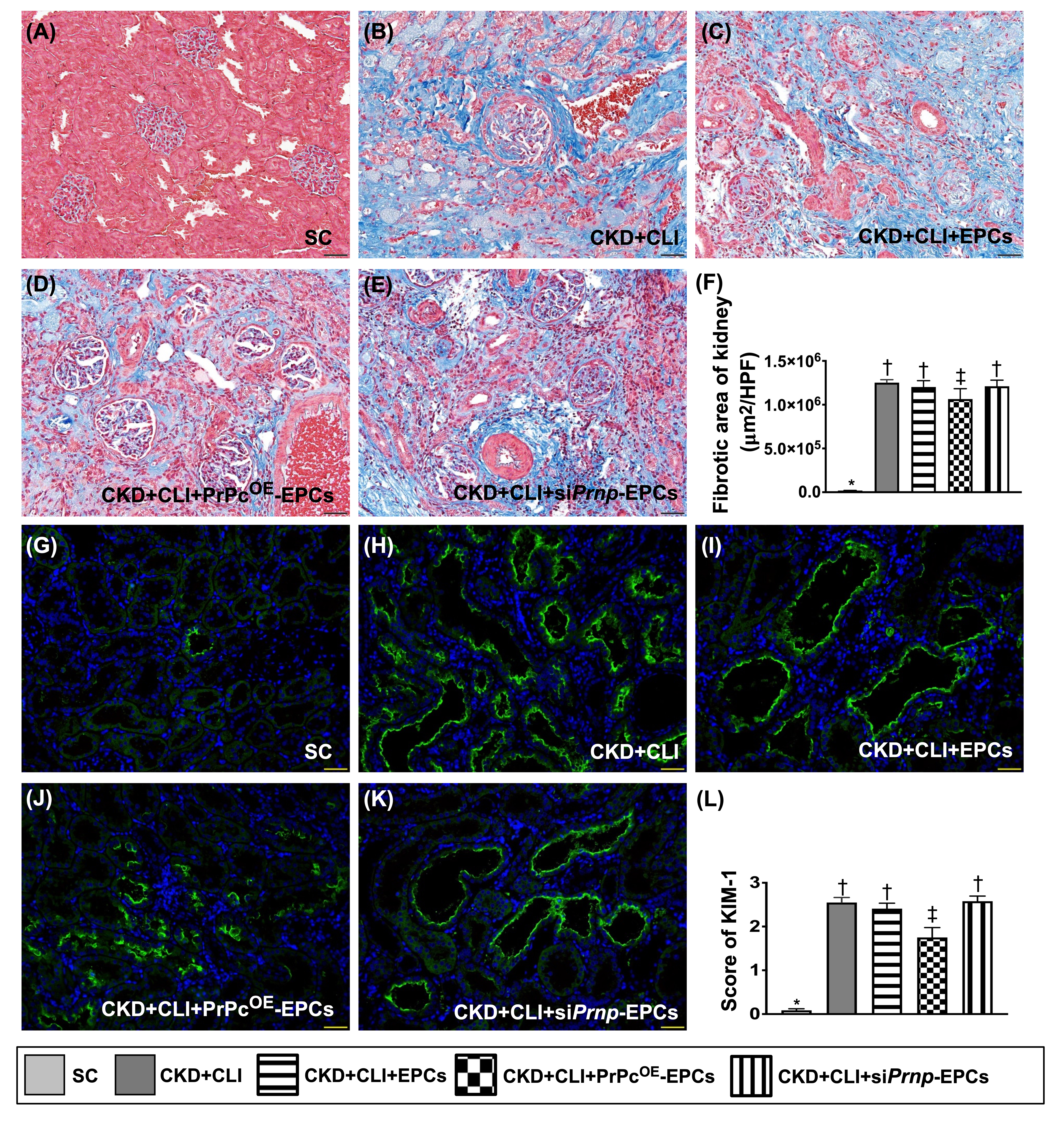

Supplement: Supplementary file 1 — Additional file 1: Fig. S1. Cellular levels of fibrosis and kidney injury biomarker in kidney parenchyma by day 42 after CKD induction. A–E illustrating the microscopic finding (200×) of Masson’s trichrome stain for identification of fibrosis in kidney parenchyma (blue color). F Analytical result of fibrotic area, * versus other groups with different symbols (†, ‡), p < 0.0001. p < 0.0001. All scale bars in right lower corner represent 50 µm. G–K Illustrating immunofluorescent (IF) microscopic finding (400×) for identification of kidney injury molecule (KIM-1) (green color). L Analytical result of expression of KIM-1, * vs. other groups with different symbols (†, ‡), p < 0.0001. p < 0.0001. Scale bars in right lower corner represent 20 µm. All statistical analyses were performed by one-way ANOVA, followed by Bonferroni multiple comparison post hoc test (n = 8 for each group). Symbols [(*, †, ‡) indicate significance (at 0.05 level). HPF = high-power field; SC = sham-operated control; CKD = chronic kidney disease; CLI = critical limb ischemia; EPCs = endothelial progenitor cells; PrPcOE-EPCs = overexpression of cellular prion protein in EPCs; siPrnp-EPCs = knockdown of cellular prion protein in EPCs [file 13287_2022_3119_MOESM1_ESM.jpg]

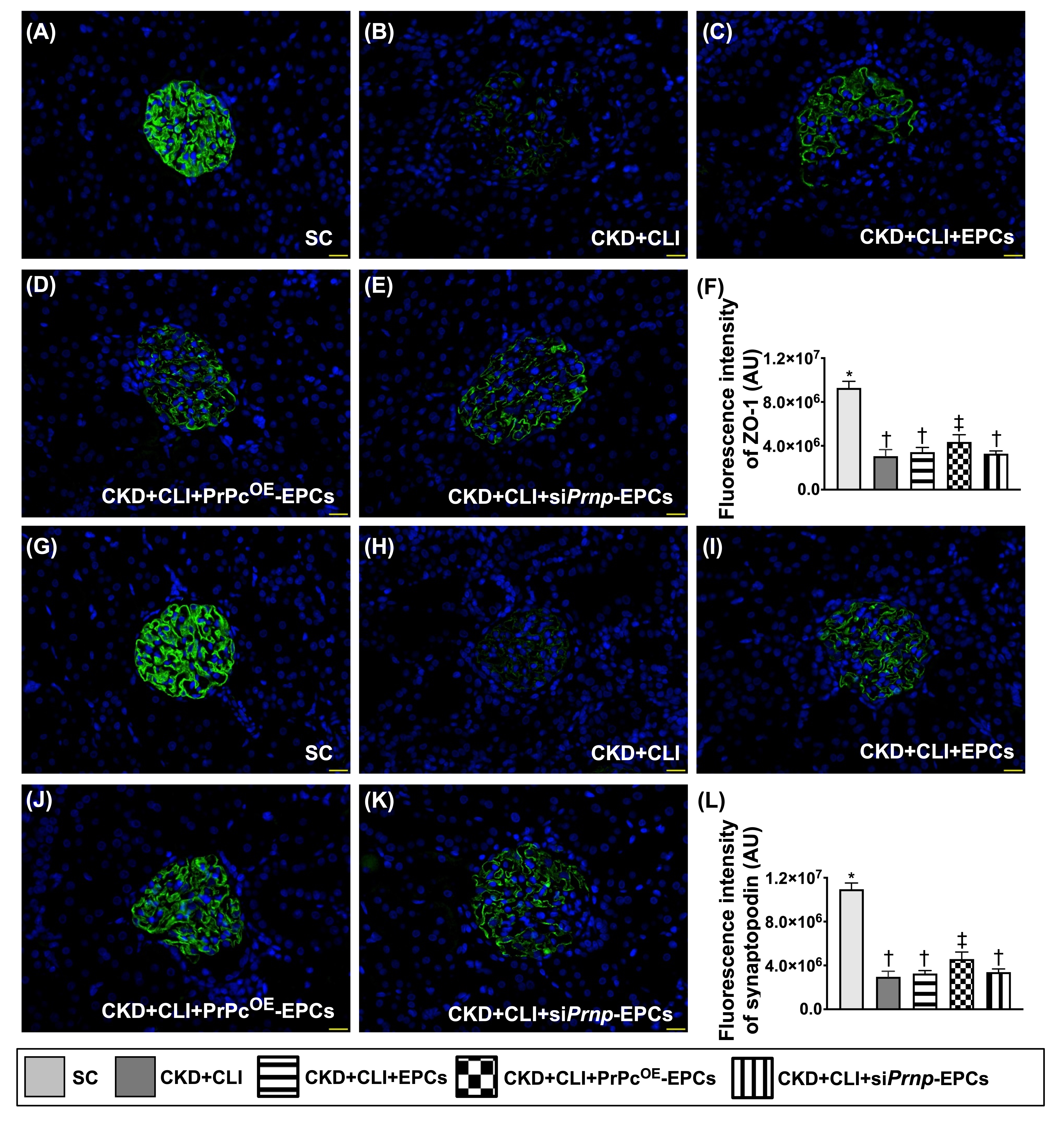

Supplement: Supplementary file 2 — Additional file 2: Fig. S2. Cellular expressions of podocyte components in glomeruli by day 42 after CKD induction. A–E Illustrating the immunofluorescent (IF) microscopic finding (400×) for identification of ZO-1 in glomeruli (green color). F Analytical result of expression of ZO-1, * versus other groups with different symbols (†, ‡), p < 0.0001. G–K Illustrating IF microscopic finding (400×) for identification of synaptopodin (green color). L Analytical result of expression of synaptopodin, * vs. other groups with different symbols (†, ‡), p < 0.0001. p < 0.0001. Scale bars in right lower corner represent 20 µm. All statistical analyses were performed by one-way ANOVA, followed by Bonferroni multiple comparison post hoc test (n = 8 for each group). Symbols [(*, †, ‡) indicate significance (at 0.05 level). SC = sham-operated control; CKD = chronic kidney disease; CLI = critical limb ischemia; EPCs = endothelial progenitor cells; PrPcOE-EPCs = overexpression of cellular prion protein in EPCs; siPrnp-EPCs = knockdown of cellular prion protein in EPCs [file 13287_2022_3119_MOESM2_ESM.jpg]
